# *QuickStats*: Percentage* of Children^†^ Aged 2–17 Years Who Wear Glasses or Contact Lenses,^§^ by Sex and Age Group — National Health Interview Survey, United States, 2019^¶^

**DOI:** 10.15585/mmwr.mm7023a4

**Published:** 2021-06-11

**Figure:**
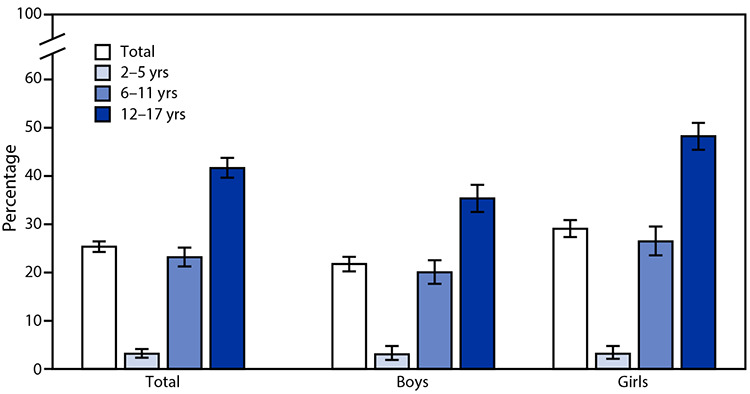
In 2019, 25.3% of children aged 2–17 years wore glasses or contact lenses, and the percentage increased with age among both boys and girls. Among boys, 3.0% wore glasses among those aged 2–5 years, 20.0% among those aged 6–11 years, and 35.3% among those aged 12–17 years. Among girls, the corresponding percentages are 3.1, 26.4, and 48.2. The percentage was higher among girls than boys overall and among those aged 6–11 years and 12–17 years, but not in the youngest age group.

**Source:** National Center for Health Statistics, National Health Interview Survey, 2019. https://www.cdc.gov/nchs/nhis.htm

